# Host–guest chemistry of mesoporous silicas: precise design of location, density and orientation of molecular guests in mesopores

**DOI:** 10.1088/1468-6996/16/5/054201

**Published:** 2015-09-25

**Authors:** Minoru Sohmiya, Kanji Saito, Makoto Ogawa

**Affiliations:** 1 Department of Earth Sciences, Waseda University, Nishiwaseda 1-6-1, Shinjuku-ku, Tokyo 169-8050, Japan; 2 Graduate School of Creative Science and Engineering, Department of Earth, Environment, and Resources Engineering, Waseda University, Nishiwaseda 1-6-1, Shinjuku-ku, Tokyo 169-8050, Japan; 3 Department of Chemical Engineering and Biomolecular Engineering, Vidyasirimedhi Institute of Science and Technology, Rayong 21210, Thailand

**Keywords:** host–guest, mesoporous silica, function, surfactant, template

## Abstract

Mesoporous solids, which were prepared from inorganic-surfactant mesostructured materials, have been investigated due to their very large surface area and high porosity, pore size uniformity and variation, periodic pore arrangement and possible pore surface modification. Morphosyntheses from macroscopic morphologies such as bulk monolith and films, to nanoscopic ones, nanoparticles and their stable suspension, make mesoporous materials more attractive for applications and detailed characterization. This class of materials has been studied for such applications as adsorbents and catalysts, and later on, for optical, electronic, environmental and bio-related ones. This review summarizes the studies on the chemistry of mesoporous silica and functional guest species (host–guest chemistry) to highlight the present status and future applications of the host–guest hybrids.

## 1. Introduction

Template syntheses have been employed as means of structural design of the materials from nanoscopic to macroscopic size regime. Surfactant aggregates (surfactant mesophases) were found to be useful to produce mesoscopic structures. The preparation method, characterization and application of mesoporous silicas and other mesostructured and mesoporous solids have been a hot topic in current materials chemistry after the discovery of mesoporous silicas (M41S and folded sheet mesoporous silica, FSM). The periodic mesostructures are prepared by the complexation of silica source with such surfactants as quaternary ammonium salts and block co-polymers as templates (structure directing agents) [1–14]. The inorganic-surfactant mesostructured materials are transformed into mesoporous solids by removal of the templates upon calcination or extraction. Since the template structures and the synthetic conditions are correlated with the mesostructures of the surfactant aggregates, the careful optimization of such synthetic parameters as concentration, temperature, pressure, duration and way of heating provides well-defined mesopores with varied shapes and inter-connection. Available pore size ranges from 1 nm to several tens of nm, keeping narrow pore size distribution. The periodically arranged pore system is another characteristic of the surfactant templated mesoporous solids, which is a requirement to achieve large surface area and porosity. The surface of mesopores can be tailored chemically and geometrically with covalently bound organic functional unit by means of direct synthesis using organosilane as a building block or post-synthetic grafting reactions [9, 10].

In addition to the structural and the compositional variations mentioned above, the morphosyntheses have mesoporous silicas more attractive for application in optics, electronics, and medical and biological fields [3, 4, 15]. Coating films, which are useful morphologies for optical, electronic and sensing devices, and self-standing films are available [16–34]. There is significant progress in designing the structural regularity and anisotropy, in addition to the microfabrication of mesoporous silica films using anisotropic substrates [35–46]. Besides the coating on flat substrates, the deposition of thin mesostructured and mesoporous silica layers on various functional powders has been achieved for constructing multi-functional core–shell particles [47–52]. The sol–gel process has been applied to obtain such macroscopic shapes as bubble, self-standing film, monolith and fiber [24, 26, 53–63]. It is also possible to control the morphology of particles precisely and systematically. Mono-dispersed spherical particles of mesoporous silicas with the particle size range of several tens of nm to a few micrometers are available [3, 4].

Thanks to the hierarchical structural and morphological variations (with periodic structure and well-defined morphology) and the possible chemical reactions in the mesostructures, a wide variety of host–guest systems have been prepared [5–7, 9–12, 64–66]. The relationship between the structure and the properties has been examined extensively. There are so many candidates of guest species to be confined in the nanospace for transformation, stabilization, storage and release, and functionalization of the nanospace, starting from ion/molecule, polymer and biopolymer to nanoparticles of metals and compounds. These guest species have been hybridized with mesoporous silicas by means of adsorption or grafting onto the pre-formed mesoporous silicas, simple impregnation and the *in situ* formation (polymerization and growth) in the mesopore. It is also possible to obtain hybrids by co-precipitation (co-polymerization) of guest species with silica sources during the synthesis of the precursors of mesoporous materials (inorganic-surfactant mesostructures). Multiple functionalization has also been done by grafting various functional groups [64, 65] and by sequential modification of functional groups bound to the surface by so-called ‘click chemistry’ [67–69].

In this review, we will overview the present status and future directions of the host–guest chemistry composed of mesoporous silicas with emphasis on the possible effects of the confinements of molecular guest species in nanospaces for the emerging new function as well as the optimization of the known functions. We have recently published a micro-review discussing the spatial distribution (location, density, and orientation) of the organic functional units/guest species in mesoporous silicas (mainly one-dimensional cylindrical mesoporous solids) [70]. In addition, some articles focused on the location of the functional units/guest species are available: on the external/internal surface [66, 71, 72], at the entrance to the mesopore [73], in the mesopore, and in the framework. In this review, we discuss the host–guest systems based on mesoporous silicas in a more general way with more detailed examples.

**Figure 1.**
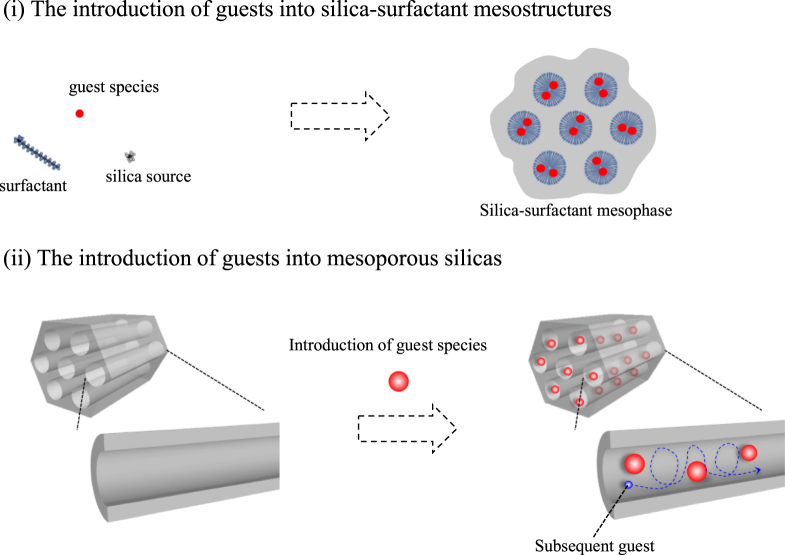
Introduction of guest species into (i) silica-surfactant mesostructures and into (ii) mesoporous silicas.

The introduction of guest species into mesostructured materials will be classified into two: (i) the introduction of guest species into silica-surfactant mesostructures and (ii) the introduction of guest species into the mesoporous solids (figure 1). One of the most important differences between the two systems is the possible remaining nanospace in the materials. In the silica-surfactant mesostructures containing guest species (i), the access and diffusion of the guest species are limited because the nanospace was filled with surfactants. If compared with the materials (i), on the other hand, the guest species (reactant in the catalysts’ application or target to be detected in the sensor application) can easily access and diffuse in the remaining nanospace (ii). In this review, we will classify the mesoporous silica-based host–guest systems from the viewpoint of the remaining pore.

## 2. Host–guest systems with filled mesostructures

There are three approaches for the introduction of guest species/functional units into the host–guest systems based on silica-surfactant mesostructures: (1) solidification from precursor solution containing soluble silica source and surfactant with dissolved functional guest species; (2) complexation of silica source with amphiphilic molecules working as supramolecular template and functional unit; and (3) polymerization of organosilanes bearing covalently bound functional units with surfactant (co-polymerization of functional organosilanes with tetraethoxysilane has also been employed). It is possible to convert the inorganic-surfactant mesostructured materials prepared by approach 3 to mesoporous ones by template removal keeping the organic functionality. The materials obtained by approaches 1 and 2 can also be transformed into mesoporous silicas after the extraction (or decomposition) of guest species. In these cases, the guest species were only used to modify the shape and size of the resulting mesopore.

### 2.1. Approach 1: solidification from precursor solution containing soluble silica source and surfactant with dissolved functional guest species

**Figure 2.**
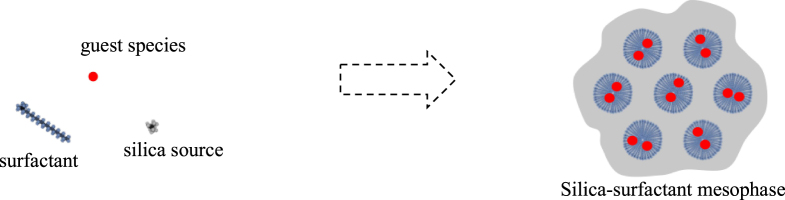
Solubilizing molecular guest species into the silica-surfactant mesophase (approach 1).

This method was the very first approach to complex dyes with silica-surfactant mesostructured materials (the image is shown in figure 2). Hydrophobic dyes such as pyrene and pyranine have been incorporated as luminescence probes into silica-surfactant mesostructured materials. Photoluminescence spectra (monomer to excimer intensity ratio) showed that pyrene was incorporated into the hydrophobic part (probably surrounded by alkyl chains of surfactant) of the silica-C_16_TAC (hexadecyltrimethylammonium chloride, hereafter abbreviated as C_16_TAC) mesostructured materials without aggregation even at a high loading level; the molar ratio of included pyrene to surfactant (C_16_TAC) was as high as 1:9, which is extremely high if compared with their solubilization into surfactant mesophases in aqueous solutions [74, 75]. Pyrene was solubilized molecularly in the silica-C_16_TAC mesostructured material and the diffusion of pyrene was restricted significantly. The mesophase of C_16_TAC aggregate was determined by the synthetic conditions (mainly the concentration and the silica to surfactant ratio) and the transformation between mesophases (for example, lamellar, hexagonal and cubic) was less plausible after the solidification with silica.

Based on the spectroscopic characteristics of the included probe molecules, the thermal phase transition and the formation mechanisms (or processes) of silica-surfactant mesostructured materials have been discussed [74–83]. The pyrene luminescence (monomer to excimer intensity ratio) as a function of temperature revealed that the microviscosity of the microenvironments surrounding the probe molecule (surfactant mesostructures immobilized by silica) was lower at lower temperature. This was also indicated by the temperature-dependent changes of the infrared absorption bands due to CH stretching vibration [75]. Thus, the photoluminescence probe study using pyrene provided a fundamental understanding of silica-C_16_TAC mesostructured materials films in the following ways: (a) inclusion capacity for the guest is comparable if compared with that for C_16_TAC aggregates in aqueous C_16_TAC solution (it will be different when applying different surfactant and guest); (b) the guest was molecularly isolated surrounded by the alkyl chain; (c) limited diffusion of guest due to the dense packing of C_16_TAC; (d) transition of gel-to-liquid crystalline states was observed; (e) the transformation between different mesophases is less plausible if compared with surfactant aggregates in solution.

The solidification from precursor solution containing soluble silica source and surfactant with dissolved functional species mentioned above has been utilized to prepare hybrids with immobilized functional dyes. Chlorophyll and phthalocyanines have been employed to construct the silica-surfactant mesostructures, where the dye aggregation was suppressed as indicated by the visible absorption spectra of the solid products [79–82]. Luminescence of pyranine was utilized to examine the formation of mesostructure during dip-coating of the precursor solution containing soluble silica source and surfactant with dissolved pyranine on substrate by solvent evaporation [77, 83].

Rhodamines have been immobilized into optically transparent solids (organic polymers and silica gels) for the application to solid-state lasing device. Hoppe *et al* has reported, for the first time, the inclusion of rhodamine B into a mesoporous silica, titanium-containing MCM-41 [80]. Marlow *et al* synthesized rhodamine 6G included silica-hexadecyltrimethylammonium bromide (C_16_TAB) mesostructured materials in the form of fiber [84] and investigated the photoemission from the fiber as a function of pump intensity utilizing second harmonic of a Nd:YAG laser (532 nm). Later on, rhodamine 6G was incorporated into silica-block copolymer ((polyethylene oxide)-(polypropylene oxide)-(polyethylene oxide), PEO-PPO-PEO, product name P123) hybrids to observe amplified spontaneous emission [85]. It should be noted here that since it is possible to obtain silica-tri-block copolymer composites as macroscopically controlled morphologies (film and monolith), accordingly the optical application and the detailed characterization are more realizable.

The patterning of mesoporous films by means of such techniques as x-ray lithography, scanning probe microscopy and imprinting methods is an important way for the miniaturization of electronic, optoelectronic and magnetic devices. Among available synthetic methods, ‘soft lithographic approaches’ have been employed to construct hierarchically designed nanoscopic materials. Trau *et al* reported patterning of mesoporous silica films by the micro-molding in capillaries (MIMIC) technique [86] to prepare a network of capillaries. Placing an elastomeric stamp in contact with a flat substrate for designed relief features, pouring into the mold an acidic precursor solution of silica-C_16_TAC and transferring the resulting materials by wicking provided the networked channel structures where cylindrical mesopores aligned parallel to the capillary walls. The patterned films containing rhodamine 6G showed amplified spontaneous emission, opening up the potential for the fabrication of integrated optical circuits [87, 88].

**Figure 3.**
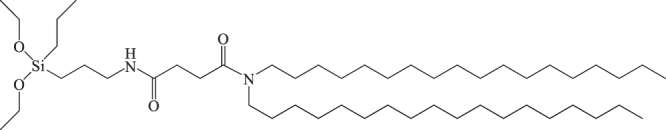
Molecular structure of oligopeptide-functionalized surfactant.

Photochromic reactions of spiropyran and spirooxazine included into silica–triblock copolymer (P123) mesostructured films, probably at the organic domain, prepared by dip-coating using dye-dissolved sol–gel precursor solution, were investigated [89]. When the dye content (or capacity) was low, thick films could be obtained, which led to an absorbance large enough to evaluate photochemical reactions by conventional instruments. The resulting materials exhibited typical photochromism of spiropyran or spirooxazine, between colorless and colored upon UV irradiation and thermal bleaching. It was claimed that the response time required for the spirooxazine to the colored form by light irradiation was very fast, making the materials attractive for optical recording (rapid recording). Ariga used oligopeptide-functionalized surfactant (figure 3) to be complexed with silica, and the resulting mesostructured silica hybrid film was shown to accommodate spiropyran [90].

For the optical and photochemical applications of the dye-containing silica-surfactant mesostructured materials, the limited capacity of the dye (guest) is not an important problem because dye generally possessed a large enough extinction coefficient to be easily detected by optical spectroscopy and in the case of film, the thickness can be tailored to obtain sufficient absorption. Thus, silica-surfactant mesostructured materials are attractive host materials for luminescent and photochromic dyes. The covalently bound (grafted) photochromic moieties on the mesopore surface of mesoporous silicas (not silica-surfactant mesostructures) have another possibility toward various photoresponsive materials, which will be discussed in the latter part of this review.

A rigidochromic dye, bathophenanthroline disulfonic acid disodium salt (ClRe(I)(CO)_3_), was embedded in MCM-41 from the precursor of MCM-41 and the states of the dye in silica-C_16_TAB was investigated as a function of pressure [91]. The luminescence-spectra changes as a function of pressure (rigidochromic property) of ClRe(I)(CO)_3_ suggested that the molecular environments in the surfactant aggregate retained some fluidity up to about 10 GPa. It was postulated that the embedded dye was protected by the rigid silica framework to probe the surrounding environments under high pressure.

**Figure 4.**
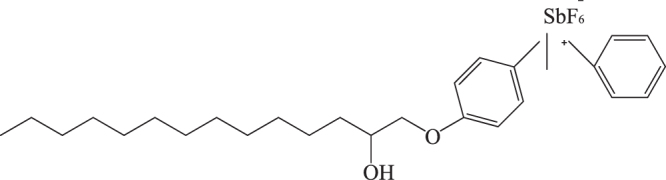
Molecular structure of diaryliodonium hexafluoroantimonate.

A molecular photoacid generator, diaryliodonium hexafluoroantimonate (figure 4), was compartmentalized into a film of silica-surfactant mesostructure [92]. The photoacid generator was dissolved in an oxyethylene surfactant, Brij 56, solution and subsequently mixed with silica sol to give the precursor solution. From the precursor solution, thin films were prepared by solvent evaporation. Acid-catalyzed siloxane condensation was promoted on the regions where ultraviolet light was irradiated, resulting in selective etching of unexposed regions.

The solubilization of various molecular species into surfactant aggregates in solutions such as micelle and vesicle as well as those on substrates (e.g. Langmuir–Blodgett film) has been studied extensively [93–95]. As to the synthetic chemistry of mesoporous materials, trimethylbenzene and alkane were solubilized into the quaternary ammonium surfactant during the preparation of MCM-41 in order to expand the pore size [96]. Polyglycerol esters of fatty acids were swollen with *n-*decane to be used as template for mesoporous silica microsphere with the interconnected pore from the exterior to interior [97]. Furthermore, surfactant mixtures with different alkylchain length were used as a mesostructure template to control pore size precisely [98]. Cationic and anionic surfactant mixture was shown to be useful for the nanostructure design [99]. Thus, the solubilization (or dissolution) of guest species into surfactant aggregate (mesophase) has been conducted not only as a way to incorporate guest species into inorganic-surfactant mesostructured materials but also from the viewpoints of designing the size, shape, and interconnection of mesopore. It is also interesting to see what will happen when the swelling agent is removed from the silica-surfactant mesostructure leaving surfactant in the products.

### 2.2. Approach 2: complexation of silica with amphiphilic molecules containing functional groups

**Figure 5.**
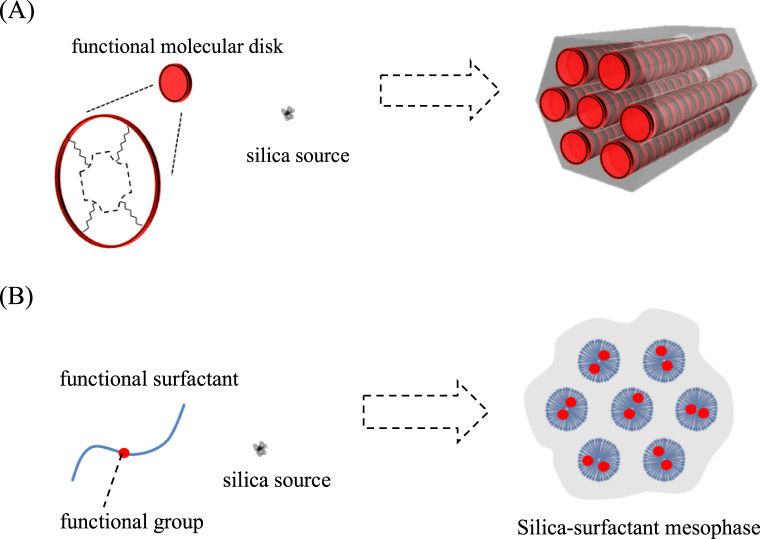
Use of amphiphilic functional compounds as supramolecular template as well as functional unit; silica–organic hybrids (approach 2).

By hybridization of amphiphilic functional compounds with silica source through sol–gel process, a mesostructured hybrid with densely packed functional units can be obtained (figure 5(a)). The possible dense packing of functional units with periodicity is an important difference between approaches 1 (incorporation of guest species into silica-surfactant mesostructured materials) and 2 (hybridization of amphiphilic functional compounds with silica). Amphiphilic metallophthalocyanine was allowed to react with tetraethoxysilane for condensation to give one-dimensional ordered stacking of molecular disk (metallophthalocyanine) [100]. Copper phthalocyanines were stacked as a single column isolated by a silica wall. One-dimensional columnar charge transfer (CT) complexes were immobilized in mesostructured silica film by sol–gel reaction of the CT complexes of an amphiphilic triphenylene donor and several acceptors [101]. The CT complexes exhibited absorption red-shift, suggesting a long-range structural order in the products, which reflects densely packed CT complex maintained by silica. Amphiphilic ferrocene, ferrocenyl-trimethylundecylammonium, was also employed to produce potentially redox active composites with electrochemical interests [102].

Polymerization of monomers in the solid-state (after complexed with silica) requires appropriate dense packing of monomer (figure 5(b)). Conjugated polymer-silica mesostructured materials have been prepared using amphiphilic diacetylene monomers [103–106]. Mesostructures (lamellar, hexagonal and cubic) of the silica-diacetylene mesostructured materials were designed by the head groups of the amphiphilic compounds, since the proximity of the diacetylene moieties strongly influences the polymerization process. The effect of the mesostructures on the polymerization of diacetylene was demonstrated by the fact that the polymerization of diacetylene was preceded for the lamellar and hexagonal mesophases by UV irradiation and subsequent heat treatment, while polymerization was inhibited for the cubic mesophase. The packing of the diacetylene moieties in the lamellar and hexagonal mesostructured materials allowed topochemical polymerization to produce colored polydiacetylene. The design of the diacetylene moiety and the hybridization with silica led to optically transparent polydiacetylene-silica mesostructured materials, which are mechanically robust if compared with conventional pristine polydiacetylene. The color of the hybrid materials changed in response to thermal, mechanical and chemical stimuli, which may be useful for their application as sensors of various targets.

One of the advantages of the hybrids of amphiphilic functional compounds is the densely packed periodic mesostructures, which made the efficient intermolecular interactions (and subsequent polymerization and CT complexation) possible. Since various amphiphilic functional molecules containing a long chain alkyl group as the hydrophobic part and an ionic or polar group as the hydrophilic part are available, approach 2 is versatile and can be combined with approaches 1 and 3 for further variation of the materials. Mesoporous materials were also obtained by the removal of the amphiphilic functional compounds (which act both as structure directing agents and functional guest species) from the hybrids (approach 2) by calcination, as exemplified by the amphiphilic metallophthalocyanine-silica hybrids, which were transformed into mesoporous silica with the pore radius of ca. 2.35 nm and the Brunauer–Emmett–Teller (BET) surface area of ca. 1000 m^2^ g^−1^ [100].

### 2.3. Approach 3: polymerization of organosilanes bearing covalently bound functional units with surfactant

**Figure 6.**
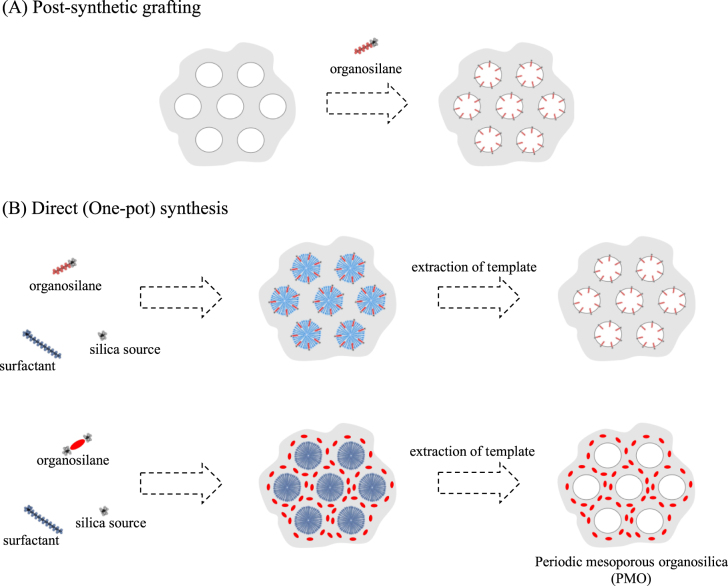
Covalent attachment of functional units onto mesostructures by (a) post-synthetic grafting and (b) direct (or one-pot) synthesis by condensation (approach 3).

Using organosilanes bearing covalently bound functional units as a silica source, mesostructured organosilicas have been also synthesized via condensation [107]: direct (one-pot) synthesis by condensation of functional organosilicon compounds: co-condensation of tetraalkoxysilanes (tetraethoxysilane (TEOS) and tetramethoxysilane (TMOS) are commonly used) and organically functionalized trialkoxysilanes (figure 6). Films of mesostructured organosilica were also available by solvent evaporation methods (evaporation induced self-assembly) [108, 109]. Polymerization of some bis(triethoxysilyl)organic precursors containing the bridging organic groups in the presence of surfactants under appropriate reaction conditions (concentration, solvent, temperature etc) resulted in the mesostructured organosilicas with periodic mesostructures where organic functional groups arrange in a periodic manner to form organic–inorganic (semi)crystalline frameworks [110].

The combination of the approaches 2 and 3 (direct synthesis using functionalized organosilane and functional amphiphilic molecule) has also been employed to construct the mesostructures for efficient electron/energy transfer between the functional unit of amphiphilic surfactant (working as supramolecular templates) and that bound to the mesopore surface [111–113].

In addition to the preparation of the host–guest hybrid materials with filled mesostructures, the direct synthesis method is useful to prepare surface functionalized mesoporous silica; by careful extraction of template subsequent to the stabilization of the mesostructures by the condensation, mesoporous organosilicas were obtained. After the pioneering study by Inagaki *et al* this class of materials was designated as periodic mesoporous organosilicas (PMO) [114]. The crystalline structures in the frameworks of PMO are determined by the structures of organic functionality bound with siloxane, so that the compositional and orientational variation of organic functionality in PMO is limited. The synthetic conditions, where inorganic condensation and template self-assembly compete, also play important roles to obtain highly periodic mesostructures. The host–guest chemistry of PMO will be discussed with some examples in section 3 of the present review.

Patterning mesostructured silica surfactants by means of pen lithography, ink-jet printing and dip-coating techniques through self-assembling of ‘ink’ have been conducted previously [115]. Organically modified mesoporous silica films were patterned by the technique from the ‘ink’ containing soluble organoalkoxysilane oligomer, which was prepared under acidic conditions and with surfactant in ethanol/water mixture. Organosilanes bearing covalently bound functional groups such as tridecafluoro-1,1,2,2-tetrahydrooctyl, aminopropyl and mercaptopropyl groups were employed. The patterning and the mesostructure formation occurred simultaneously during drying the ‘ink’. From the patterned mesostructured films, surfactant was successfully removed by pyrolysis or solvent extraction without degrading the organic moiety covalently bound to the Si atom, indicating the thermal and chemical stability of the organic moiety which is covalently bound to the silica network. The states and functions of the remaining organic groups in the resulting mesoporous materials have been discussed.

Viologen moiety was covalently bound to the siloxane network and the photoreduction of the viologen was observed [116–118]. The sample was synthesized from viologen-bound trialkoxysilane and C_16_TAB. Photochemical reaction was conducted for the as-synthesized materials containing C_16_TAB. Though the mechanism of the photoreduction was not explained clearly, the photoreduction of viologen in the mesostructured materials are worth investigating further for the construction of efficient photoinduced electron transfer systems partly for future artificial photosynthesis application. The surfactant was removed to obtain viologen-containing porous organosilica with the BET surface area of ca. 900 m^2^ g^−1^; however, photochemical reaction was not reported for the porous organosilica.

Self-assembly of long chain alkyltrialkoxysilane was conducted, where alkyl group was bound to Si atom, and during the condensation, they self-assembled into periodic mesostructures [107, 119–121]. Various alkylalkoxysilanes have been synthesized and polymerized to obtain periodic mesostructures, which has been reviewed previously [107, 119].

## 3. Spatial distribution (location, density and orientation) of the guest species for designing open mesopore

**Figure 7.**
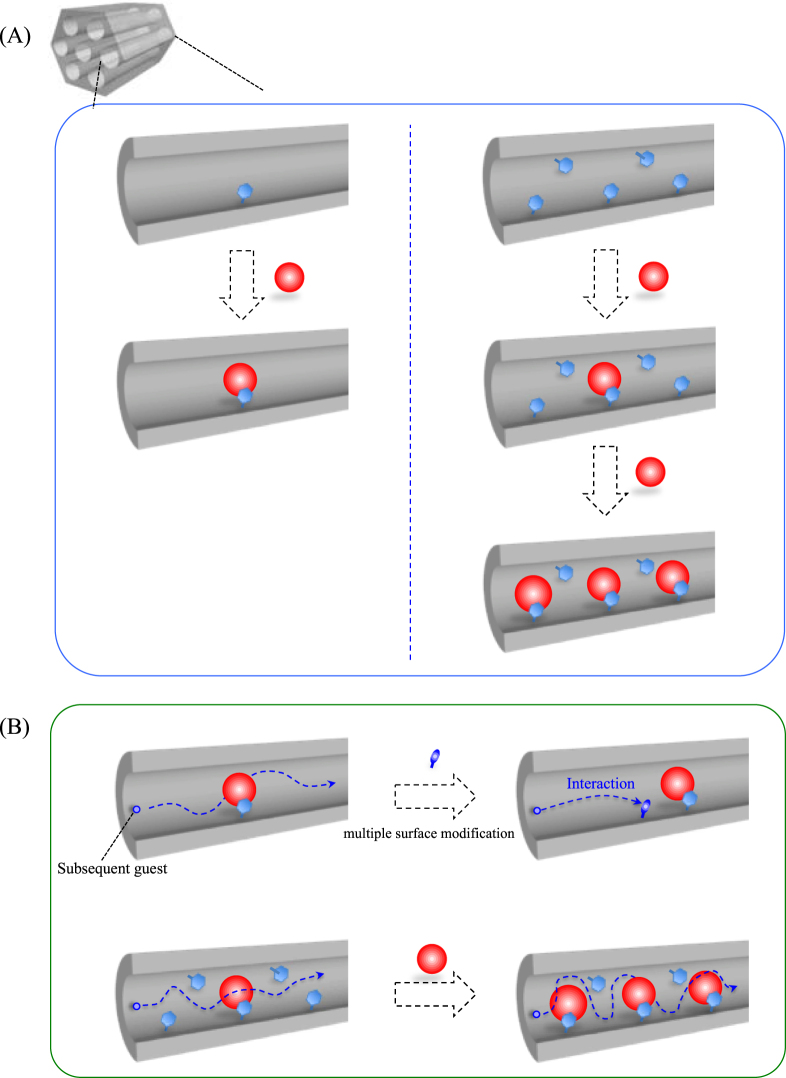
(a) Schematic drawings of the variation of the remaining nanospaces after guest immobilization in surface-modified mesoporous materials. (b) Schematic drawings of the diffusion of subsequent guest (adsorbate, reactant or products) in the remaining nanospaces.

Silica-surfactant mesostructured materials were discussed in section 2 as functional host–guest systems. For their applications as catalysts, sensors, reservoirs and carriers of drugs, the guest species (functional units) immobilized in mesoporous materials are more attractive from the viewpoints of reactant access, diffusion and molecular selectivity in catalyst and sensor application, large capacity and controlled release for reservoir and drug delivery applications and so on. In order to modify the characteristics of the host–guest hybrids, the spatial distribution (location, density and orientation) as well as the diffusion of the guest species are parameters to be concerned (figure 7). Accordingly, various functional units (guest species) have been immobilized in mesoporous silicas: in the silica framework, on the mesopore surface and on the external surface of the materials.

### 3.1. Guest species in the silica framework: Heteroelement-containing mesoporous silicas and periodic mesoporous organosilicas

The introduction of such heteroelements as Al, Ti and Fe into mesoporous silicas has been conducted to impart catalytic functions on the mesoporous materials and to control the surface chemical properties [122, 123]. The shape and size of the mesopore are not altered significantly by the heteroelement introduction into the silica framework under the carefully determined synthetic conditions, as indicated by the nitrogen adsorption/desorption isotherms. Moreover, the transmission electron microscopy and nitrogen adsorption/desorption data provide information on the mesoporous structures by the same way used for the siliceous mesoporous materials. If compared with the introduction of heteroelements onto the mesopore surface by the adsorption from solution, the direct synthesis is characterized by simple preparation (one-pot synthesis) and possible controlled location of the doped heteroelement in the silica framework.

Titanium-containing mesoporous silicas have been synthesized by direct synthetic or post synthetic methods using surfactant and molecular precursors containing titanium. The states of the doped titanium have been examined for the optimization of catalytic performances [124–136]. The immobilization of titanium dioxide particles inside the mesopore is also a topic of interest and is described later. Various spectroscopic techniques including UV–vis absorption, photoluminescence and x-ray absorption fine structure (XAFS) have been employed to evaluate the states of the doped titanium species. As an example, titanium-containing nanoporous silica films were prepared by the solvent evaporation method. UV–vis absorption, photoluminescence and XAFS spectroscopy showed that the doped titanium exists as an isolated one with tetrahedrally coordinated oxygen in the silica framework (the tetrahedrally coordinated isolated titanium is a known a photocatalytically active species) [133–136]. The titanium-containing nanoporous silica film photocatalytically converted CO_2_ and H_2_O into CH_4_ and CH_3_OH with high selectivity for the formation of CH_3_OH, showing the characteristic reactivity of the charge transfer excited complexes of the tetrahedral coordinated titanium oxide species. The heteroelement-containing mesoporous silicas are possible catalysts for various reactions. It should be noted here that the attachment of molecular or clustered titanium species to the mesopore using titanium complexes through impregnation as well as coordination changes the shape of the original mesopore to some extent. Detailed characterization of the remaining pore (shape and size) after the post-synthetic introduction of titanium species was not well understood.

Another example of the functional units in the mesoporous silica frameworks (silica wall) is PMOs [110, 114]. Coprecipitation of bis(triethoxysilyl)organic precursors and surfactants (template) results in the mesostructured organosilicas where the organic groups take periodic arrangements to form organic–inorganic semi-crystalline frameworks. Mesoporous organosilicas can be obtained after the template extraction. Recently, the immobilization of molecular catalysts into PMO was investigated toward artificial photosyntheses [137–140]. Photoinduced energy transfer from the organic group occluded inside the framework of organosilica (PMO) with periodic manner to the molecular catalyst inside the mesopore (*antenna* function) was utilized for the reduction of carbon dioxide. To put it plainly, the PMO acted not only as a scaffold to accommodate molecular catalysts but also a photosensitizer to construct a light-harvesting system. The mesopore provided the space for the immobilization of catalysts and for the diffusion of reactants and products.

### 3.2. Guest species inside the mesopore

**Figure 8.**
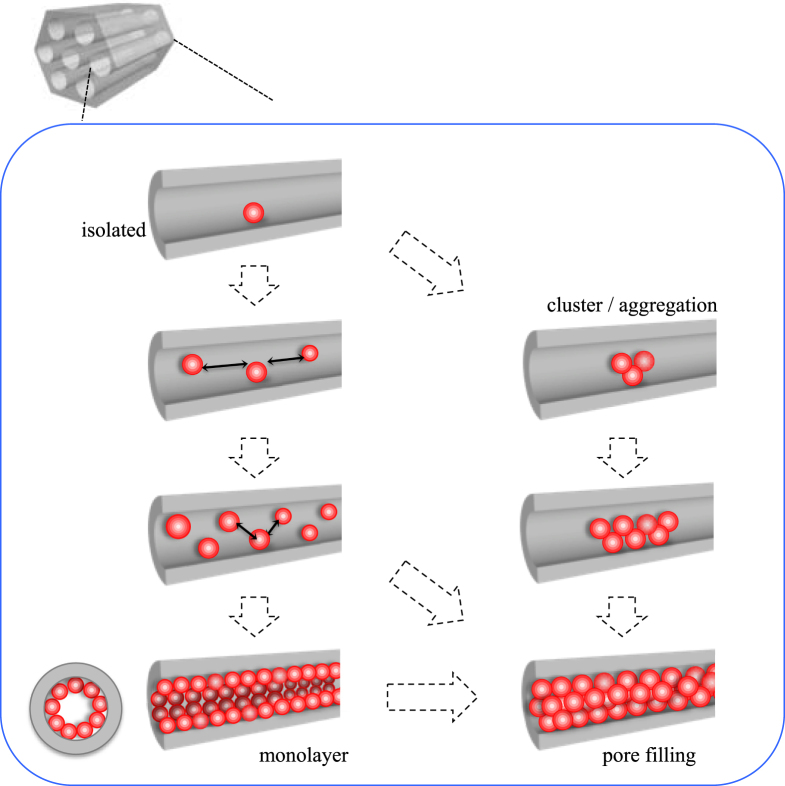
Schematic drawings of the variation in the guest species included in a mesopore.

Besides the functional units in the silica framework, guest species may exist on the mesopore surface. In this case, the mesopore can be partially occupied by the guest species to provide nanospace surrounded by the silica and the guest, or can be fully occupied by the guest as schematically shown in figure 8. The guest species can be located inside the mesopore as isolated molecules, monolayer, cluster (molecular aggregate), or completely filling state. The ways to attach guest species on the mesopore surfaces can be classified into two: (1) adsorption of nonionic or cationic species on mesopore; and (2) covalent binding of functional unit, for example, using functionalized alkoxysilane. For the covalent bindings, both direct (one-pot) synthetic and post-synthetic grafting methods are applicable as mentioned above.

**Figure 9.**
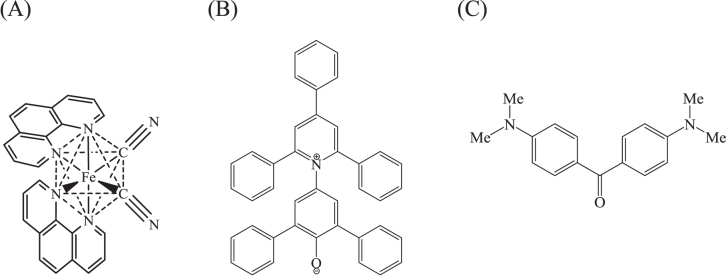
Molecular structures of (a) dicyanobis(1,10-phenanthroline)iron(II), (b) 2,6-diphenyl-4-(2,4,6-triphenyl-1-pyridinio)phenolate, and (c) Michler’s ketone.

The adsorption of solvatochromic dyes onto MCM-41 was conducted by Spange *et al*, in order to probe the polarity of the mesopore surface [141]. The spectroscopic characteristics of the solvatochromic dyes, 2,6-diphenyl-4-(2,4,6-triphenyl-1-pyridinio)phenolate, dicyanobis(1,10)phenanthroline)iron(II) and Michler’s ketone (figure 9) varied reflecting the dye–surface interactions. It was proposed that the polarity of the mesopore surface of MCM-41 can be quantitatively described by the following three terms: the dipolarity/polarizability, the hydrogen-bond-donating ability and the hydrogen-bond-accepting ability. The dipolarity/polarizability of the mesopore surface was temperature independent, while the hydrogen-bond-donating ability was larger at lower temperature. It should be noted here that the surface properties of the mesopore are determined by not only the three terms (the dipolarity/polarizability, the hydrogen-bond-donating ability and the hydrogen-bond-accepting ability) but also other parameters. It is required to carefully discuss the surface under controlled experimental conditions (temperature, pressure, humidity etc).

The adsorption of basic species has been conducted through electrostatic interactions with the silanol group on the pore surface [12]. The states of the dye can be discussed based on the spectroscopic characteristics (intensity, wavelength, decay and anisotropy) even at very low loading. The photoluminescence of coumarin 540 adsorbed in MCM-41 by thermal diffusion in a vacuum was investigated to see luminescence red-shift, indicating the possible interactions between the dye and the mesopore surface [142]. The photophysical properties of 2,4,6-triphenylpyrylium and para-substituted tritylium ions encapsulated in MCM-41 was investigated [143] to show that the luminescence and transient triplet–triplet excited-state absorption spectra of 2,4,6-triphenylpyrylium ion in MCM-41 are similar to those in solution, while a dramatic enhancement of the emission intensity was observed for 2,4,6-triphenylpyrylium ion in MCM-41. Such spectroscopic study (both absorption and luminescence) is useful to probe the chemistry of surface and can be applied for other mesoporous materials. In general, very low dye loading was favorable to study the host–guest interactions to avoid the effects of guest–guest interactions. It is also interesting to investigate the effects of the surface modification, while it is not straightforward to discuss the differences due to such additional factors as guest–guest interactions and pore size effects on the states of the guest species.

As to the host–guest systems where the guest exists only inside the mesopore, the amount and distribution of guests are topics of interests for the materials characteristics. Depending on the amount (or concentration) of the guest in the mesopore, the states of the guest vary from molecularly isolated to aggregated, and finally completely filled in the mesopore. By the adsorption of guest from solutions with different concentration, the amount of the guest occluded can be tuned as a result of chemical equilibrium. For the adsorption of cationic porphin into MCM-41, red-shifted absorption bands were observed at high loading level to indicate the aggregation of the guests adsorbed [144]. The state of the aluminum tris(oxine) complex occluded changed during wet-impregnation of the complex into mesoporous silicas [145, 146]. In addition to the possible pore size effects on the states of guest [147–149], the loading amount is a possible parameter to affect the states (aggregation or isolation) of the guest. Polymers including dendrimers have been incorporated into mesopores by infiltration [150–152]. Size exclusion of dendrimers for the inclusion into a mesopore was achieved to show the possible separation of the dendrimer generation. By the infiltration of dendrimer containing metallic nanoparticles, the nanoparticles can be introduced into the mesopore with the dendrimer [152]. In such simple impregnation (infiltration) driven host–guest systems, where the host–guest interactions are relatively weak, there is a wide range of variations in the state of the guest. In addition to the host–guest chemical interactions, the size matching between the pore and the guest and the amount of the guest species included are possible concerns for the distribution (density and orientation) and diffusion of the guest species. Synthetic conditions (sample pre-treatment, solvent, reaction temperature, atmosphere, etc) are also important to affect the states of the guest species.

On the other hand, guest–guest interactions can be utilized to design such functions as polymerization, charge transfer and intermolecular reaction, including photoinduced ones. The photosensitized isomerization of stilbene by photoexcited 2,4,6-triphenylpyrylium cation co-adsorbed in MCM-41 was reported [153]. The introduction of the dyes into the mesopores was conducted by the impregnation from CH_2_Cl_2_ solution of the dye and by the *in situ* synthesis of the dye in the pore. The chemical yield for the cis-stilbene was remarkably higher than those in homogeneous systems, showing the possible role of the mesopore of MCM-41 for controlled inter- and intra-molecular reactions as a result of appropriate placement (or controlled distance between two dyes) of two species (sensitizer and reactant) within the mesopore. The location of two dyes, intermolecular separation and diffusion, is thought to be concerned with the confinement effect on the photosensitization. In addition to the acceleration of the rates of energy transfer, the confinement of pyrene in mesoporous silica results in the changes of the equilibrium and kinetics of bimolecular reaction (excimer formation) in comparison to that in solution, possibly because of the modified diffusion and probability of the reaction [154].

**Figure 10.**
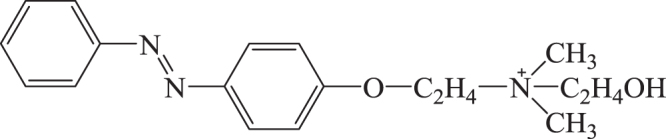
Molecular structure of *p*-(*ω*-dimethyl-hydroxyethylammonioethoxy)-azobenzene bromide (AZ^+^).

The introduction of a heteroelement into the siliceous framework can be applied to control the distribution of guest species. Cationic dyes such as methylene blue or cationic cyanine derivatives were adsorbed onto siliceous mesoporous silica films from solutions [155], while the cationic azobenzene derivative, *p*-(*ω*-dimethyl-hydroxyethylammonioethoxy)-azobenzene bromide (abbreviated as AZ^+^; figure 10), was not adsorbed effectively [156]. The effective adsorption of AZ^+^ without aggregation was achieved by employing the mesoporous silica containing aluminum in the framework. There is a linear relationship between the amounts of AZ^+^ adsorbed and the Al contents of the host mesoporous silica films, showing that the added Al substitutes Si to generate the negative charge (or acidic site) quantitatively for the adsorption of AZ^+^. To evaluate the states of Al in the mesoporous silica powders or bulk solids, various techniques including temperature-programmed desorption and ^27^Al nuclear magnetic resonance spectroscopy have been employed. However, these techniques are difficult to be applied for the thin mesoporous silica films mainly due to the detection limit. The adsorption of basic dyes with large absorption coefficient and efficient photoluminescence quantum yield is a possible method for the quantitative evaluation of the acidic and cation exchange ability of the thin films [157, 158]. The adsorbed AZ^+^ in the mesopore exhibited photochemical isomerization by UV and visible light irradiation. The ratio of the cis*-*isomer formed by UV irradiation at the photostationary state at room temperature was estimated to be ca. 70%. Considering the density of the guest species, there is enough free space for AZ^+^ to isomerize even at the maximum loading level in Al-containing mesoporous silica films. Thus, it was demonstrated that the mesoporous silica films are possible nano-vessels for efficient photochemical reactions which required geometrical changes.

The introduction of Ca into the mesoporous aluminosilica powder resulted in the effective adsorption of an acidic dye, Lithol Rubin B [159], while the driving force for the adsorption is not well explained. The light-fastness properties of the composites varied depending on the presence of Ca and the ways for the Ca introduction. Various dyes have been incorporated to test the stability [160–163]. Aluminium- or iron-containing mesoporous silicas were used for the stabilization of flavylium dye [162, 163]. In addition to the possible role of the interactions between the pore surface and the dye, it was suggested that oxygen diffusion in the mesopore is concerned with the dye stability. From this respect, the remaining open space after the adsorption of the dyes should be optimized. Into the remaining open space, solution can be infiltrated as shown by the example of the clay–polymer system. Smectite–water soluble polymer was used to stabilize a cationic dye efficiently compared with that included in pristine polymer and that intercalated in pristine smectite [164].

Photoinduced electron transfer from the adsorbed dyes (N-alkylphenothiazines or meso-tetraphenylporphyrin) to the mesoporous solids was investigated [165–167]. The efficiency of the photoinduced electron transfer and the stability of the photochemically formed species were varied depending on the heteroelements (titanium, aluminum and vanadium) in the framework of mesoporous silicas, moreover, the pore size and the alkylchain length of the N-alkylphenothiazines, indicating that not only the chemical properties of the surface but also steric configuration (the size of pore and adsorbed species) should be considered for controlling the photoinduced intermolecular reactions.

The surface modification with the silane coupling agents containing amino or cyano groups enabled the incorporation of the rare-earth complex [C_5_H_5_NC_16_H_33_][Eu(TTA)_4_] into mesoporous silicas. The adsorbed complex exhibited higher UV stability and longer excited state lifetime [168, 169]. Europium-doped yttria was prepared in SBA-15 with a pore diameter of more than 5 nm by impregnating aqueous rare-earth nitrate solution and subsequent calcination, while the optical properties of the resulting products were reported to be disappointing from the viewpoint of luminescence quantum yield [170]. The surface modification of mesoporous silica with 1,4-butanediol suppressed the pheophytinization of chlorophyll a during the introduction [171, 172]. Among possible mesopore surface functionalization, the ‘lizard template’ proposed by Ariga Zhang *et al* is a unique from the viewpoint of creating a nanopore with designed functional units on the pore surface [173]. This was achieved by co-condensation of organosilicon compounds containing alanine residue with tetraethoxysilane and subsequent cleavage of the alkyl ‘tail’ by selective hydrolysis of the ester. As a result of the ‘tail’ removal, an open pore with the surface alanine residue was obtained. The porous materials were employed as a reactor artificial enzyme for the catalytic capability of unhydrolyzed materials on acetalization of a ketone [174].

Covalent bonding of photofunctional guest species to the mesopore surface has been conducted by copolymerization of tetraethoxysilane and dye-bound alkoxysilane in the presence of surfactant and subsequent removal of the surfactant [175–179]. The co-condensation of TEOS and 3-(2,4-dinitrophenylamino)propyltriethoxysilane in the presence of C_16_TAB resulted in the transparent yellow thin films and monoliths of dye bound silica-surfactant mesostructured materials. When this process was conducted within the regular voids of a colloidal crystal composed of monosized polystyrene spheres, hierarchically ordered dye-functionalized mesostructured silicas formed [176]. Ganschow *et al* synthesized dye (azo and rhodamine dyes) bound mesoporous silicas by a microwave-assisted hydrothermal reaction [178]. It was reported that the rapid microwave heating led the significant reduction of the synthesis time to prevent dye degradation during the preparation.

Fluorescein-bound mesoporous silica film was prepared by co-condensation of fluorescein isothiocyanate with 3-aminopropyltriethoxysilane and a block copolymer (F127) [179]. The resulting film showed pH-dependent photoluminescence intensity. The photoluminescence intensity changed in a few seconds upon the change in the pH. The fast response was attributed to the high porosity and the free space large enough for the diffusion of H^+^ (OH^−^) of the film. A similar pH sensor based on mesoporous organosilicas with 5,6-carboxyfluorescein as a pH sensing unit was obtained using patterned self-assembled monolayer with a self-assembling ‘ink’ approach [115].

The replacement with the surfactant of silica-surfactant mesostructured materials is a way to incorporate the guest species in a mesopore [180]. Cationic species can be included into the mesoporous silica by replacement of cationic surfactant from silica-surfactant mesostructures. Poly(*p*-phenylenevinylene) was immobilized into mesoporous system by the template displacement and *in situ* polymerization [181].

We have been interested in the adsorption of tris(2,2’-bipyridine)ruthenium(II) complex cation (abbreviated as [Ru(bpy)_3_]^2+^) into mesoporous silicas from the viewpoint of the spatial distribution of [Ru(bpy)_3_]^2+^ inside the mesopore, how to control the spatial distribution and how the spatial distribution affects the performances of the resulting host–guest systems. The host–guest systems composed of [Ru(bpy)_3_]^2+^ and mesoporous silicas have been investigated for possible applications for catalyst, electrode, etc [182–184]. The adsorption of [Ru(bpy)_3_]^2+^ onto mesoporous silicas with the average pore sizes of a few nanometers was conducted from dimethylformamide solution of [Ru(bpy)_3_]^2+^ chloride [185, 186]. By changing the concentration of the solutions, the amount of [Ru(bpy)_3_]^2+^ adsorbed was controlled up to less than 1 mass% at a maximum. Dehydration of [Ru(bpy)_3_]^2+^-mesoporous silicas induced the aggregation of [Ru(bpy)_3_]^2+^ ions adsorbed, which was shown by the efficient luminescence self-quenching. The luminescence was intensified dramatically upon hydration of the samples, suggesting that the aggregated [Ru(bpy)_3_]^2+^ ions were de-aggregated to be dispersed molecularly in the mesopore. Thus, the states of the guest species are affected not only by the host–guest interactions but also by the co-adsorbed species. This is an important difference between the host–guest systems with (described in section 2 of the present review) and without (described here in section 3) open nanospaces for the easy access and diffusion of other various molecular species.

[Ru(bpy)_3_]^2+^-containing FSM (which was formed by the reaction of a layered silicate kanemite with alkyltrimethylammonium salts, with the hexagonally arranged cylindrical mesoporous structure similar to MCM-41) was used as a photocatalyst to oxidize benzene to phenol [183]. For the optimized performance of this kind of molecular catalyst immobilized in porous solids, such characteristics as the access and diffusion of reactants and products, and the efficiency of activation (photo-excitation) are important concerns. Host–guest interactions and guest–guest interactions of the immobilized molecular catalysts should be carefully tuned in order to achieve the designed distribution of the catalytically active sites on the mesopore surface. As a result of the controlled distribution, which will be related to the efficiency of the excitation and stability of the excited states, and the access and diffusion of reactants and products, the efficiency of the reaction will be optimized. Taking these points into consideration, we have extended the host–guest systems composed of [Ru(bpy)_3_]^2+^ and mesoporous silicas using modified mesoporous silicas [187–190]. The surface density (or concentration) of the molecular catalyst ([Ru(bpy)_3_]^2+^) has been controlled within 0.8 *μ* mol m^−2^ mesopore surface or 1.0 mol L^−1^ mesopore pore volume not only by the varied loading of molecular catalyst but also by the surface density (or concentration) of immobilized functional unit (sulfonated phenyl group), which interacts with the molecular catalysts). As mentioned above, these characteristics are important to design the open space for the access and diffusion of reactants and products and the efficiency of activation (photo-excitation).

The application of mesoporous silica as proton exchange membrane has been a topic of interest [191–194]. In order to control the useful proton conducting properties, various host–guest systems have been developed. Such acidic functionalities as imidazole [195], sulfonic acid [194, 196, 197], phosphonic acid [198] and hetelopolyacid (HPA) [199–205] have been immobilized in the mesoporous silicas. Among acidic functionalities immobilized in mesoporous silicas, HPA is one of the most promising candidates. HPAs are known ionic conductors and hybridization has been conducted for proton exchange membrane [206] and catalyst [207] applications. Jiang *et al* reported the application of HPA immobilized mesoporous silica hybrids as a fuel cell separator. They applied two methods for the sample preparation; one is one-step method where precursors of HPA, TEOS and structure directing agent (P123) was solidified by condensation [202–205], and the other is impregnation of HPA into pre-synthesized mesoporous silica [199–201]. In the latter case, the impregnation of HPA into mesoporous silica was conducted under a vacuum, i.e. vacuum-assisted impregnation (VIM). It was claimed that the VIM has advantages to achieve homogeneous distribution of HPA in the mesopore if compared with those prepared by the conventional impregnation method. The immobilization method is also important to control the spatial distribution of guest species in the mesopore.

The spatial distribution (location, density and orientation) of polar guest species in the nanospace plays important roles for the nonlinear optical property. Nonlinear optics (NLO) comprises the interaction of light with matter to produce a new light field which is different from incident light in wavelength or phase, including frequency mixing processes (sum harmonic generation, optical parametric amplification, optical parametric generation), optical Kerr effect etc. For the production of NLO materials based on the host–guest systems, it is required to control the orientation of the guest molecule at a microscopic level. *p*-Nitroaniline (pNA) was introduced into MCM-41 and boron-doped MCM-41 to obtain second harmonic generation (SHG) materials (SHG is a nonlinear optical process which converts two input photons into a single output photon at twice the input frequency) [208]. The SHG activity was evaluated for the powder sample immersed in silicone grease to be deposited as a thin film between two glass plates. The products exhibited SHG, indicating that the pNA molecules arranged in a manner without centrosymmetry in the cylindrical mesopore. For the NLO applications of the host–guest systems, the density of guest species is important from the viewpoints of the stronger response as well as the anisotropic orientation of the dye in the one-dimensional mesopore. For the practical application of the NLO device based on mesoporous materials, samples in the form of thick films and bulk samples with appropriate optical quality are desirable.

**Figure 11.**
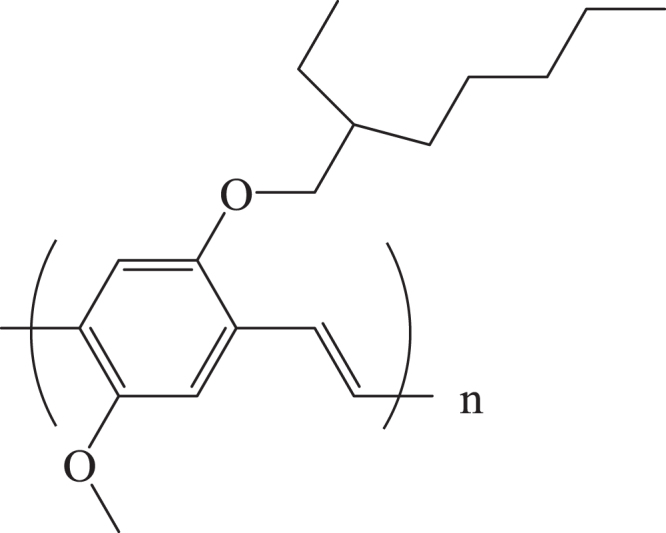
Molecular structures of poly[2-methoxy-5(2′-ethoxyhexloxy)-1,4-phenylenevinylene].

The adsorption of a monomer and subsequent polymerization of the monomer in the mesopore have been investigated. Poly(aniline) and poly(acrylonitrile) were synthesized in MCM-41 with the pore diameter of 2.5 nm [209–211]. Nguyen *et al* reported the introduction of poly[2-methoxy-5(2′-ethoxyhexloxy)-1,4-phenylenevinylene] (figure 11) into a mesoporous silica with 2.2 nm pore diameter [212, 213]. The host mesoporous silica was prepared under magnetic field to align the pore direction, in order to control the ordering of the incorporated the poly(phenylenevinylene). Polymer was incorporated from solution and the resulting material was index-matched in a glycerol/propanol mixture to suppress scattering for the optical measurements. The dynamics of the excited states were studied by using steady-state and time-resolved polarized luminescence spectroscopy. The excitations migrate unidirectionally from aggregated and randomly oriented polymer chains located outside the pore to isolated and aligned polymer chains within the pore. It was shown that the intra-chain energy migration occurred more slowly than inter-chain Förster energy transfer.

### 3.3. Hierarchical structural design

Anisotropy of the guest species included in mesoporous materials has also been a topic of interest toward three dimensionally controlled geometry of the host–guest system. This kind of research became available after the progresses of hierarchically controlled mesostructure preparation and characterization [35, 36, 41–46, 214–217]. One-dimensionally oriented cylindrical mesopore in the macroscopically oriented film has been reported [36, 41, 214]. Mesoporous silica films with a preferential orientation of mesopores were obtained using a continuous-flow reactor as evidenced from scanning electron microscopy images of the substrate surface [45]. Bulk mesostructured silica with high degree of macroscopic alignment was synthesized by utilizing capillary flow or parallel plate shearing [23]. Ordered mesoporous silica films with the oriented mesochannels perpendicular to the film plane were obtained by electrochemically assisted method [215, 216], by applying magnetic fields [46] and by simple deposition on a substrate from Stöber solution [217]. Taking advantage of the one-dimensionally oriented cylindrical mesopores, various guest species, including dyes [218], semiconductor polymers [219–223], and nanoparticles [224, 225], have been introduced into them to show anisotropic properties. Anisotropic cyanine dye aggregation has been observed in oriented mesoporous silica film [218]. Due to the strong tendency of the dye to form aggregate, the concentration of the dye is not an important parameter for the orientational order in this case. When the dye loading was low, the dyes form aggregate in some part of the mesopore. The spatial distribution of the aggregate may be a next target to be investigated because the communication of the aggregates (confined electron) with adjacent dye aggregates or the pore surface can be used to obtain hierarchically designed hybrid materials.

In addition to the films, pore geometries of well-defined (and anisotropic) particles have been a topic of interest. As to the mesoporous silica spherical particles, Yano and Fukushima reported the radially arranged one-dimensional pore [226], which can be used for the anisotropic guest inclusion. The mesopore interconnected with micropore or interpore-neck [227–229] made the discussion on the location and diffusion of the guest species more complicated. In addition, well-defined platy- and disk-shaped hollow particles became available [230, 231]. Not only the shape and the size of the dual pore systems, but also the surface can be modified selectively in order to construct hierarchically designed host–guest systems with possible guest distribution and diffusion.

## 4. Conclusions and future perspectives

Due to the ability of mesopores to accommodate various kinds of guest species, the variations in the host–guest systems are huge, which accounts for the rich materials chemistry on this topic. The structures are determined by the selection of host and guest and the interfacial design of host–guest interactions. The location of guest species varies: at the external/internal surface, inside the pore, at the entrance to the pore, and within the framework. For each possible location, the homogeneity, density and orientation of the guest species should be considered in multi-length scales. The spatial distribution (location, density and orientation) and host–guest interactions affect the diffusion and stability of the guest species, which are worth investigating further [232]. The conditions to prepare and to characterize the host–guest materials are basically important for the reliable and reproducible results as well as possible optimized performances.

Possible applications of host–guest complexes of mesoporous silicas proposed include catalyst, adsorbent, drug carrier, sensor and photonics. The spatial distribution of guest species (or functional units) can be designed to initiate desired reactions in catalyst applications, to bind target species strongly in adsorbent applications, to accommodate drug molecules in drug delivery applications, to react with target molecules selectively in sensing applications, etc. Accompanied by the accommodation of functional units/guest species, the remaining nanospaces after the attachment of functional units or the inclusion of guest species should be considered geometrically, topologically and chemically for efficient diffusion of reactants and products (before and after the catalytic reaction) and adsorbates (during adsorption), for controlled up-take and release of drug (in drug delivery) and for possible molecular recognition (in sensing). In addition, the dynamic behavior of the functional units on the pore surface, which change its conformation and position to fit the resulting host–guest complexes in more stable states (induced fits), has been examined recently [233–235].

Besides the organic compounds, inorganic nano-species such as heteropolyacids, semiconductor nanoparticles and nanocarbons have been encapsulated in the mesoporous silicas. The host–guest complexes of mesoporous and mesostructured transition metal oxides and sulfides, metals, carbons and polymers have an opportunity to which mesoporous silicas do not have access. Thus, utilizing a supramolecular templating strategy can provide a huge variety of nanostructured materials from diverse building units including both host and guest. The unlimited variations and possibilities in the construction of the host–guest systems derived from the mesoporous and mesostructured materials will be investigated for the practical applications, where precise design of the structures (both static and dynamic) of host–guest complexes are of great importance.

## References

[C1] Ariga K, Vinu A, Yamauchi Y, Ji Q M, Hill J P (2012). Bull. Chem. Soc. Japan.

[C2] Ruiz-Hitzky E, Aranda P, Darder M, Ogawa M (2011). Chem. Soc. Rev..

[C3] Wu S H, Mou C Y, Lin H P (2013). Chem. Soc. Rev..

[C4] Shiba K, Shimura N, Ogawa M (2013). J. Nanosci. Nanotechnol..

[C5] Moller K, Bein T (1998). Chem. Mater..

[C6] Ciesla U, Schuth F (1999). Microporous Mesoporous Mater..

[C7] Scott B J, Wirnsberger G, Stucky G D (2001). Chem. Mater..

[C8] Taguchi A, Schuth F (2005). Microporous Mesoporous Mater..

[C9] Stein A, Melde B J, Schroden R C (2000). Adv. Mater..

[C10] Stein A (2003). Adv. Mater..

[C11] Ogawa M (2002). J. Photochem. Photobiol. C.

[C12] Tran-Thi T H, Dagnelie R, Crunaire S, Nicole L (2011). Chem. Soc. Rev..

[C13] Lebeau B, Innocenzi P (2011). Chem. Soc. Rev..

[C14] Soler-Illia G J, Azzaroni O (2011). Chem. Soc. Rev..

[C15] Mann S, Ozin G A (1996). Nature.

[C16] Ogawa M (1994). J. Am. Chem. Soc..

[C17] Ogawa M (1996). Chem. Commun..

[C18] Ogawa M (1997). Langmuir.

[C19] Ogawa M, Igarashi T, Kuroda K (1997). Bull. Chem. Soc. Japan.

[C20] Ayral A, Balzer C, Dabadie T, Guizard C, Julbe A (1995). Catal. Today.

[C21] Dabadie T, Ayral A, Guizard C, Cot L, Lacan P (1996). J. Mater. Chem..

[C22] Klotz M, Albouy P A, Ayral A, Menager C, Grosso D, van der Lee A, Cabuil V, Babonneau F, Guizard C (2000). Chem. Mater..

[C23] Melosh N A, Davidson P, Feng P, Pine D J, Chmelka B F (2001). J. Am. Chem. Soc..

[C24] Anderson M T, Martin J E, Odinek J G, Newcomer P P, Wilcoxon J P (1997). Microporous Mater..

[C25] Martin J E, Anderson M T, Odinek J, Newcomer P (1997). Langmuir.

[C26] Ryoo R, Ko C H, Cho S J, Kim J M (1997). J. Phys. Chem. B.

[C27] Ogawa M, Masukawa N (2000). Microporous Mesoporous Mater..

[C28] Yang H, Coombs N, Sokolov I, Ozin G A (1996). Nature.

[C29] Yang H, Coombs N, Dag O, Sokolov I, Ozin G A (1997). J. Mater. Chem..

[C30] Yang H, Coombs N, Ozin G A (1998). J. Mater. Chem..

[C31] Ruggles J L, Holt S A, Reynolds P A, Brown A S, Creagh D C, White J W (1999). Phys. Chem. Chem. Phys..

[C32] Balkus K J, Scott A S, Gimon-Kinsel M E, Blanco J H (2000). Microporous Mesoporous Mater..

[C33] Clark T, Ruiz J D, Fan H Y, Brinker C J, Swanson B I, Parikh A N (2000). Chem. Mater..

[C34] Hozumi A, Sugimura H, Hiraku K, Kameyama T, Takai O (2000). Chem. Mater..

[C35] Yamaguchi A, Uejo F, Yoda T, Uchida T, Tanamura Y, Yamashita T, Teramae N (2004). Nat. Mater..

[C36] Miyata H, Kuroda K (2000). Chem. Mater..

[C37] Miyata H, Kuroda K (1999). Adv. Mater..

[C38] Miyata H, Kuroda K (1999). J. Am. Chem. Soc..

[C39] Miyata H, Kuroda K (1999). Adv. Mater..

[C40] Miyata H, Kuroda K (1999). Chem. Mater..

[C41] Miyata H, Suzuki T, Fukuoka A, Sawada T, Watanabe M, Noma T, Takada K, Mukaide T, Kuroda K (2004). Nat. Mater..

[C42] Yang P (2000). Science.

[C43] Tolbert S H, Firouzi A, Stucky G D, Chmelka B F (1997). Science.

[C44] Fukumoto H, Nagano S, Kawatsuki N, Seki T (2006). Chem. Mater..

[C45] Hillhouse H W, Okubo T, vanEgmond J W, Tsapatsis M (1997). Chem. Mater..

[C46] Yamauchi Y, Sawada M, Noma T, Ito H, Furumi S, Sakka Y, Kuroda K (2005). J. Mater. Chem..

[C47] Ogawa M, Shimura N, Ayral A (2006). Chem. Mater..

[C48] Kato R, Shimura N, Ogawa M (2008). Chem. Lett..

[C49] Nakamura K J, Ide Y, Ogawa M (2011). Mater. Lett..

[C50] Ide Y, Koike Y, Ogawa M (2011). J. Colloid Interface Sci..

[C51] Ogawa M, Naito D, Shimura N (2007). Chem. Lett..

[C52] Shimura N, Ogawa M (2007). J. Colloid Interface Sci..

[C53] Ogawa M, Kikuchi T (1998). Adv. Mater..

[C54] Ogawa M, Yamamoto N (1999). J. Porous Mater..

[C55] Nagamine S, Endo A, Nakaiwa M, Nakane T, Kurumada K, Tanigaki M (2001). Microporous Mesoporous Mater..

[C56] Roser S J, Patel H M, Lovell M R, Muir J E, Mann S (1998). Chem. Commun..

[C57] Attard G S, Glyde J C, Goltner C G (1995). Nature.

[C58] Fowler C E, Lebeau B, Mann S (1998). Chem. Commun..

[C59] Melosh N A, Lipic P, Bates F S, Wudl F, Stucky G D, Fredrickson G H, Chmelka B F (1999). Macromolecules.

[C60] Tolbert S H, Schaffer T E, Feng J L, Hansma P K, Stucky G D (1997). Chem. Mater..

[C61] Huo Q S, Feng J L, Schuth F, Stucky G D (1997). Chem. Mater..

[C62] Ogawa M, Yamamoto N (1999). Langmuir.

[C63] Bruinsma P J, Kim A Y, Liu J, Baskaran S (1997). Chem. Mater..

[C64] Shylesh S, Thiel W R (2011). ChemCatChem.

[C65] Yu C, He J (2012). Chem. Commun..

[C66] Argyo C, Weiss V, Bräuchle C, Bein T (2014). Chem. Mater..

[C67] De Los C O (2012). Chem. Mater..

[C68] Burglova K, Noureddine A, Hodacova J, Toquer G, Cattoen X, Wong Chi M M (2014). Chem. Eur. J..

[C69] Noureddine A, Lichon L, Maynadier M, Garcia M, Gary-Bobo M, Zink J I, Wong Chi Man M, Cattoën X (2015). Nanoscale.

[C70] Ogawa M, Saito K, Sohmiya M (2015). Eur. J. Inorg. Chem..

[C71] Kecht J, Schlossbauer A, Bein T (2008). Chem. Mater..

[C72] Cauda V, Schlossbauer A, Kecht J, Zurner A, Bein T (2009). J. Am. Chem. Soc..

[C73] Song N, Yang Y W (2015). Chem. Soc. Rev..

[C74] Ogawa M (1995). Langmuir.

[C75] Ogawa M, Igarashi T, Kuroda K (1998). Chem. Mater..

[C76] Ohtaki M, Inata K, Eguchi K (1998). Chem. Mater..

[C77] Sellinger A, Weiss P M, Nguyen A, Lu Y F, Assink R A, Gong W L, Brinker C J (1998). Nature.

[C78] Zhou H S, Honma I (1998). Chem. Lett..

[C79] Hata H, Kimura T, Ogawa M, Sugahara Y, Kuroda K (2000). J. Sol-Gel Sci. Technol..

[C80] Hoppe R, Ortlam A, Rathousky J, SchulzEkloff G, Zukal A (1997). Microporous Mater..

[C81] Zhou H S, Sasabe H, Honma I (1998). J. Mater. Chem..

[C82] Ganschow M, Wöhrle D, Schulz-Ekloff G (1999). J. Porphyrins Phthalocyanines.

[C83] Huang M H, Dunn B S, Soyez H, Zink J I (1998). Langmuir.

[C84] Marlow F, McGehee M D, Zhao D Y, Chmelka B F, Stucky G D (1999). Adv. Mater..

[C85] Wirnsberger G, Stucky G D (2000). Chem. Mater..

[C86] Trau M, Yao N, Kim E, Xia Y N, Whitesides G M, Aksay I A (1997). Nature.

[C87] Yang P D (2000). Science.

[C88] Scott B J, Wirnsberger G, McGehee M D, Chmelka B F, Stucky G D (2001). Adv. Mater..

[C89] Wirnsberger G, Scott B J, Chmelka B F, Stucky G D (2000). Adv. Mater..

[C90] Ariga K (2004). Chem. Rec..

[C91] Wu J J, Abu-Omar M M, Tolbert S H (2001). Nano Lett..

[C92] Doshi D A (2000). Science.

[C93] Clint J H 1992 Surfactant Aggregation New York: Blackie

[C94] Goddard E D and Anathapadmanabhan K P 1993 Interactions of Surfactants with Polymers and Proteins Boca Raton, FL: CRC Press

[C95] Ulman A 1992 An Introduction to Ultrathin Organic Films San Diego, CA: Academic

[C96] Beck J S (1992). J. Am. Chem. Soc..

[C97] Kapoor M P, Vinu A, Fujii W, Kimura T, Yang Q, Kasama Y, Yanagi M, Juneja L R (2010). Microporous Mesoporous Mater..

[C98] Khushalani D, Kuperman A, Coombs N, Ozin G A (1996). Chem. Mater..

[C99] Chen F X, Huang L M, Li Q Z (1997). Chem. Mater..

[C100] Kimura M, Wada K, Ohta K, Hanabusa K, Shirai H, Kobayashi N (2001). J. Am. Chem. Soc..

[C101] Okabe A, Fukushima T, Ariga K, Aida T (2002). Angew. Chem. Int. Edn..

[C102] Honma I, Zhou H S (1998). Adv. Mater..

[C103] Lu Y (2001). Nature.

[C104] Aida T, Tajima K (2001). Angew. Chem. Int. Edn..

[C105] Peng H S, Tang J, Yang L, Pang J B, Ashbaugh H S, Brinker C J, Yang Z Z, Lu Y F (2006). J. Am. Chem. Soc..

[C106] Ikegame M, Tajima K, Aida T (2003). Angew. Chem. Int. Edn..

[C107] Kuroda K, Shimojima A, Kawahara K, Wakabayashi R, Tamura Y, Asakura Y, Kitahara M (2014). Chem. Mater..

[C108] Lu Y F, Fan H Y, Doke N, Loy D A, Assink R A, LaVan D A, Brinker C J (2000). J. Am. Chem. Soc..

[C109] Dag O, Yoshina-Ishii C, Asefa T, MacLachlan M J, Grondey H, Coombs N, Ozin G A (2001). Adv. Funct. Mater..

[C110] Mizoshita N, Tani T, Inagaki S (2011). Chem. Soc. Rev..

[C111] Minoofar P N, Hernandez R, Chia S, Dunn B, Zink J I, Franville A C (2002). J. Am. Chem. Soc..

[C112] Minoofar P, Hernandez R, Franville A C, Chia S Y, Dunn B, Zink J I (2003). J. Sol-Gel Sci. Technol..

[C113] Johansson E, Zink J I (2007). J. Am. Chem. Soc..

[C114] Inagaki S, Guan S, Ohsuna T, Terasaki O (2002). Nature.

[C115] Fan H, Lu Y, Stump A, Reed S T, Baer T, Schunk R, Perez-Luna V V, Lopez G P, Brinker C J (2000). Nature.

[C116] Álvaro M, Ferrer B, Fornés V, García H (2001). Chem. Commun..

[C117] Álvaro M, Ferrer B, Fornés V, García H (2003). ChemPhysChem.

[C118] Álvaro M, Ferrer B, García H, Hashimoto S, Hiratsuka M, Asahi T, Masuhara H (2004). ChemPhysChem.

[C119] Hoffmann F, Froba M (2011). Chem. Soc. Rev..

[C120] Shimojima A, Sugahara Y, Kuroda K (1998). J. Am. Chem. Soc..

[C121] Shimojima A, Umeda N, Kuroda K (2001). Chem. Mater..

[C122] Stein A, Holland B (1996). J. Porous Mater..

[C123] Tuel A (1999). Microporous Mesoporous Mater..

[C124] Tanev P T, Chibwe M, Pinnavaia T J (1994). Nature.

[C125] Maschmeyer T, Rey F, Sankar G, Thomas J M (1995). Nature.

[C126] Zhang W H, Froba M, Wang J L, Tanev P T, Wong J, Pinnavaia T J (1996). J. Am. Chem. Soc..

[C127] Luan Z H, Kevan L (1997). J. Phys. Chem. B.

[C128] Ikeue K, Yamashita H, Anpo M (1999). Chem. Lett..

[C129] Zhang S G, Fujii Y, Yamashita K, Koyano K, Tatsumi T, Anpo M (1997). Chem. Lett..

[C130] Xu Y M, Langford C H (1997). J. Phys. Chem. B.

[C131] Corma A, Garcia H, Navarro M T, Palomares E J, Rey F (2000). Chem. Mater..

[C132] Yamashita H, Yoshizawa K, Ariyuki M, Higashimoto S, Anpo M, Che M (2001). Chem. Commun..

[C133] Ogawa M, Ikeue K, Anpo M (2001). Chem. Mater..

[C134] Ikeue K, Nozaki S, Ogawa M, Anpo M (2002). Catal. Lett..

[C135] Ikeue K, Nozaki S, Ogawa M, Anpo M (2002). Catal. Today.

[C136] Shioya Y, Ikeue K, Ogawa M, Anpo M (2003). Appl. Catal. A.

[C137] Inagaki S, Ohtani O, Goto Y, Okamoto K, Ikai M, Yamanaka K, Tani T, Okada T (2009). Angew. Chem. Int. Edn..

[C138] Takeda H, Ohashi M, Tani T, Ishitani O, Inagaki S (2010). Inorg. Chem..

[C139] Yui T, Takeda H, Ueda Y, Sekizawa K, Koike K, Inagaki S, Ishitani O (2014). ACS Appl. Mater. Interfaces.

[C140] Yamamoto Y, Takeda H, Yui T, Ueda Y, Koike K, Inagaki S, Ishitani O (2014). Chem. Sci..

[C141] Spange S, Zimmermann Y, Graeser A (1999). Chem. Mater..

[C142] Gu G, Ong P P, Li Q T (1999). J. Phys. D: Appl. Phys..

[C143] Cano M L, Cozens F L, Garcia H, Marti V, Scaiano J C (1996). J. Phys. Chem..

[C144] Yoshida A, Kakegawa N, Ogawa M (2003). Res. Chem. Intermed..

[C145] Tagaya M, Ogawa M (2006). Chem. Lett..

[C146] Tagaya M, Ogawa M (2008). Phys. Chem. Chem. Phys..

[C147] Roduner E 2006 Nanoscopic Materials—Size Dependent Phenomena Cambridge: RSC Publishing

[C148] Morishige K, Shikimi M (1998). J. Chem. Phys..

[C149] Schreiber A, Ketelsen I, Findenegg G H (2001). Phys. Chem. Chem. Phys..

[C150] Angelatos A S, Wang Y, Caruso F (2008). Langmuir.

[C151] Diaz I, Garcia B, Alonso B, Casado C M, Moran M, Losada J, Perez-Pariente J (2003). Chem. Mater..

[C152] Huang W, Kuhn J N, Tsung C K, Zhang Y, Habas S E, Yang P, Somorjai G A (2008). Nano Lett..

[C153] Corma A, Fornes V, Garcia H, Miranda M A, Sabater M J (1994). J. Am. Chem. Soc..

[C154] Thomas A, Polarz S, Antonietti M (2003). J. Phys. Chem. B.

[C155] Ogawa M, Ishikawa H, Kikuchi T (1998). J. Mater. Chem..

[C156] Ogawa M, Mori J, Kuroda K (2000). Stud. Surf. Sci. Catal..

[C157] Ogawa M, Kuroda K, Mori J (2000). Chem. Commun..

[C158] Ogawa M, Kuroda K, Mori J (2002). Langmuir.

[C159] Shibata M, Hotta H, Suzuki T, Valange S, Gabelica Z (1999). Chem. Lett..

[C160] Gabelica Z, Valange S, Shibata M, Hotta H, Suzuki T (2001). Microporous Mesoporous Mater..

[C161] Laguna H, Loera S, Ibarra I A, Lima E, Vera M A, Lara V (2007). Microporous Mesoporous Mater..

[C162] Kohno Y, Tsubota S, Shibata Y, Nozawa K, Yoda K, Shibata M, Matsushima R (2008). Microporous Mesoporous Mater..

[C163] Kohno Y, Senga M, Shibata M, Yoda K, Matsushima R, Tomita Y, Maeda Y, Kobayashi K (2011). Microporous Mesoporous Mater..

[C164] Sohmiya M, Omata S, Ogawa M (2012). Polym. Chem..

[C165] SungSuh H M, Luan Z H, Kevan L (1997). J. Phys. Chem. B.

[C166] Krishna R M, Prakash A M, Kevan L (2000). J. Phys. Chem. B.

[C167] Luan Z H, Bae J Y, Kevan L (2000). Chem. Mater..

[C168] Xu Q H, Li L S, Li B, Yu J H, Xu R R (2000). Microporous Mesoporous Mater..

[C169] Xu Q H, Li L S, Liu X S, Xu R R (2002). Chem. Mater..

[C170] Sauer J, Marlow F, Spliethoff B, Schuth F (2002). Chem. Mater..

[C171] Murata S, Hata H, Kimura T, Sugahara Y, Kuroda K (2000). Langmuir.

[C172] Murata S, Furukawa H, Kuroda K (2001). Chem. Mater..

[C173] Zhang Q M, Ariga K, Okabe A, Aida T (2004). J. Am. Chem. Soc..

[C174] Otani W, Kinbara K, Zhang Q, Ariga K, Aida T (2007). Chem. Eur. J..

[C175] Yoshikawa T, Nakamura T, Kuroda K, Ogawa M (2002). Bull. Chem. Soc. Japan.

[C176] Lebeau B, Fowler C E, Hall S R, Mann S (1999). J. Mater. Chem..

[C177] Lebeau B, Fowler C E, Mann S, Farcet C, Charleux B, Sanchez C (2000). J. Mater. Chem..

[C178] Ganschow M, Wark M, Wohrle D, Schulz-Ekloff G (2000). Angew. Chem. Int. Edn..

[C179] Wirnsberger G, Scott B J, Stucky G D (2001). Chem. Commun..

[C180] Yonemitsu M, Tanaka Y, Iwamoto M (1997). Chem. Mater..

[C181] Kelly T L, Che S P, Yamada Y, Yano K, Wolf M O (2008). Langmuir.

[C182] Liu C B, Ye X K, Wu Y (1996). Catal. Lett..

[C183] Fujishima K, Fukuoka A, Yamagishi A, Inagaki S, Fukushima Y, Ichikawa M (2001). J. Mol. Catal. A: Chem..

[C184] Villemure G, Pinnavaia T J (1999). Chem. Mater..

[C185] Ogawa M, Nakamura T, Mori J, Kuroda K (2000). J. Phys. Chem. B.

[C186] Ogawa M, Nakamura T, Mori J I, Kuroda K (2001). Microporous Mesoporous Mater..

[C187] Sohmiya M, Ogawa M (2011). Microporous Mesoporous Mater..

[C188] Sohmiya M, Ogawa M (2011). Bull. Chem. Soc. Japan.

[C189] Ogawa M, Kuroda K, Nakamura T (2002). Chem. Lett..

[C190] Sohmiya M, Sugahara Y, Ogawa M (2007). J. Phys. Chem. B.

[C191] Hogarth W H J, Diniz da C J C, Lu G Q (2005). J. Power Sources.

[C192] Xiong L, Nogami M (2006). Chem. Lett..

[C193] Li H B, Nogami M (2002). Adv. Mater..

[C194] Athens G L, Ein-Eli Y, Chmelka B F (2007). Adv. Mater..

[C195] Marschall R, Sharifi M, Wark M (2009). Microporous Mesoporous Mater..

[C196] Mikhailenko S, Desplantier-Giscard D, Danumah C, Kaliaguine S (2002). Microporous Mesoporous Mater..

[C197] Marschall R, Bannat I, Feldhoff A, Wang L Z, Lu G Q, Wark M (2009). Small.

[C198] Jin Y G, Qiao S Z, Xu Z P, Yan Z M, Huang Y N, da Costa J C D, Lu G Q (2009). J. Mater. Chem..

[C199] Lu S, Wang D, Jiang S P, Xiang Y, Lu J, Zeng J (2010). Adv. Mater..

[C200] Zeng J, Jiang S P (2011). J. Phys. Chem. C.

[C201] Zhou Y, Yang J, Su H, Zeng J, Jiang S P, Goddard W A (2014). J. Am. Chem. Soc..

[C202] Tang H, Pan M, Lu S, Lu J, Jiang S P (2010). Chem. Commun..

[C203] Tang H, Pan M, Jiang S P (2011). Dalton Trans..

[C204] Lu J, Tang H, Lu S, Wu H, Jiang S P (2011). J. Mater. Chem..

[C205] Zeng J, Shen P K, Lu S, Xiang Y, Li L, De Marco R, Jiang S P (2012). J. Membr. Sci..

[C206] Rao P M, Wolfson A, Kababya S, Vega S, Landau M (2005). J. Catal..

[C207] Dias A S, Pillinger M, Valente A A (2006). Microporous Mesoporous Mater..

[C208] Kinski I, Gies H, Marlow F (1997). Zeolites.

[C209] Wu C G, Bein T (1994). Chem. Mater..

[C210] Wu C G, Bein T (1994). Science.

[C211] Wu C G, Bein T (1994). Science.

[C212] Nguyen T Q, Wu J, Tolbert S H, Schwartz B J (2001). Adv. Mater..

[C213] Nguyen T Q, Wu J, Doan V V, Schwartz B J, Tolbert S H (2000). Science.

[C214] Miyata H (2007). Microporous Mesoporous Mater..

[C215] Walcarius A, Sibottier E, Etienne M, Ghanbaja J (2007). Nat. Mater..

[C216] Goux A, Etienne M, Aubert E, Lecomte C, Ghanbaja J, Walcarius A (2009). Chem. Mater..

[C217] Teng Z, Zheng G, Dou Y, Li W, Mou C Y, Zhang X, Asiri A M, Zhao D (2012). Angew. Chem. Int. Edn..

[C218] Fukuoka A, Miyata H, Kuroda K (2003). Chem. Commun..

[C219] Wu J J, Gross A F, Tolbert S H (1999). J. Phys. Chem. B.

[C220] Molenkamp W C, Watanabe M, Miyata H, Tolbert S H (2004). J. Am. Chem. Soc..

[C221] Cadby A J, Tolbert S H (2005). J. Phys. Chem. B.

[C222] Martini I B, Craig I M, Molenkamp W C, Miyata H, Tolbert S H, Schwartz B J (2007). Nat. Nanotechnol..

[C223] Ding L H, Li W Z, Wang Q H, Sun Q Q, He Y Y, Su B (2014). Chem. Eur. J..

[C224] Shan F, Lu X M, Zhang Q, Su B, Lu Q H (2012). Langmuir.

[C225] Suzuki T, Miyata H, Noma T, Kuroda K (2008). J. Phys. Chem. C.

[C226] Yano K, Fukushima Y (2004). J. Mater. Chem..

[C227] Ryoo R, Ko C H, Kruk M, Antochshuk V, Jaroniec M (2000). J. Phys. Chem. B.

[C228] Innocenzi P, Malfatti L, Kidchob T, Falcaro P (2009). Chem. Mater..

[C229] Zhao D Y, Yang P D, Margolese D I, Chmelka B F, Stucky G D (1998). Chem. Commun..

[C230] Machida S, Yoshida T, Hashimoto R, Ogawa M (2014). J. Colloid Interface Sci..

[C231] Hashimoto R, Tsuji Y, Ogawa M (2012). J. Mater. Sci..

[C232] Ogawa M, Saito K, Sohmiya M (2014). Dalton Trans..

[C233] Mihai S, Cazacu A, Arnal-Herault C, Nasr G, Meffre A, van der Lee A, Barboiu M (2009). New J. Chem..

[C234] Le Duc Y, Gilles A, Mihai S, Rouessac V, Tingry S, Barboiu M (2013). Chem. Commun..

[C235] Barboiu M (2015). Eur. J. Inorg. Chem..

